# Single-crystalline chromium silicide nanowires and their physical properties

**DOI:** 10.1186/s11671-015-0776-8

**Published:** 2015-02-06

**Authors:** Han-Fu Hsu, Ping-Chen Tsai, Kuo-Chang Lu

**Affiliations:** Department of Materials Science and Engineering, National Cheng Kung University, No.1, University Rd, Tainan, 701 Taiwan; Center for Micro/Nano Science and Technology, National Cheng Kung University, No.1, University Rd, Tainan, 701 Taiwan

**Keywords:** CVD, Chromium silicide nanowires, Field emission, Ferromagnetic property

## Abstract

In this work, chromium disilicide nanowires were synthesized by chemical vapor deposition (CVD) processes on Si (100) substrates with hydrous chromium chloride (CrCl_3_ · 6H_2_O) as precursors. Processing parameters, including the temperature of Si (100) substrates and precursors, the gas flow rate, the heating time, and the different flow gas of reactions were varied and studied; additionally, the physical properties of the chromium disilicide nanowires were measured. It was found that single-crystal CrSi_2_ nanowires with a unique morphology were grown at 700°C, while single-crystal Cr_5_Si_3_ nanowires were grown at 750°C in reducing gas atmosphere. The crystal structure and growth direction were identified, and the growth mechanism was proposed as well. This study with magnetism, photoluminescence, and field emission measurements demonstrates that CrSi_2_ nanowires are attractive choices for future applications in magnetic storage, photovoltaic, and field emitters.

**Electronic supplementary material:**

The online version of this article (doi:10.1186/s11671-015-0776-8) contains supplementary material, which is available to authorized users.

## Background

Recently, transition metal silicide nanowires have been widely studied [1-9] for their utilization in semiconductor device technologies. Low-resistivity silicides, such as TiSi_2_, CoSi_2_, and NiSi, have been applied for interconnection in CMOS devices [10]. The group of refractory semiconducting silicides, composed of silicon and metals, have different physical properties that are useful and importantly meaningful. Among them, semiconducting silicides, such as CrSi_2_ and ß-FeSi_2_, with a narrow energy gap (0.1 to 0.9 eV) have been extensively investigated for their potential use in silicon-integrated optoelectronic devices [11] such as LEDs [12,13] and IR detectors [14]. In particular, CrSi_2_ is a narrow bandgap (0.35 eV) semiconductor [15-17], offering applications in the Schottky barrier solar cell technology [18]. Hexagonal CrSi_2_ with a C40-type structure has a high melting point and excellent resistance to oxidation, deformation, and stretching, being considered to be a potential structural material for aerospace and energy generation industries [19]. Additionally, it is a thermoelectric conversion component that could be applied to generate electric power at high temperatures [20]; the figure of merit (ZT) of CrSi_2_ has been measured to be 0.25 at 900 K [21]. CrSi_2_ also has good field emission with relatively low work function (3.9 eV) [22] as compared with generally studied field emission materials such as CNTs (5 eV) [23] and ZnO (5.3 eV) [24]. With excellent intrinsic properties of CrSi_2_, one-dimensional CrSi_2_ nanowires are expected to improve field emission performances by bulk and thin film CrSi_2_. Though there have been some previous studies on CrSi_2_ nanowires [25-28], two special aspects can be found in this research. Firstly, we conducted a more systematic study on the influences of each processing parameter on growth. Secondly, we provided a low-cost and simple method to synthesize high-quality CrSi_2_ nanowires with very good physical properties.

## Methods

In our experiments, we synthesized chromium disilicide nanowires with chemical vapor deposition (CVD) processes. Single-crystal Si (001) wafers, the native oxide of which was etched by BOE solution, were substrates. The metal source was from hydrous chromium chloride (CrCl_3_ · 6H_2_O) powders, and the flow gas is Ar gas (99.99%). The CrCl_3_ · 6H_2_O powders were put in the upstream zone of the furnace, where the temperature ranged from 700°C to 800°C, while the silicon (001) substrates were put in the downstream zone with the same temperature range. During the growth process, with oxygen environment, CrSi_2_ nanowires may transform to be CrSi_2_(core)/SiO_2_(shell) nanowires due to oxidation. To understand what factors influence the growth of chromium disilicide nanowires, we varied reaction time and temperatures of substrates and the metal source. Scanning electron microscopy (SEM), X-ray diffraction (XRD), and transmission electron microscopy (TEM) studies were conducted for morphology observation and structure identification of the nanowires. Additionally, physical properties, including magnetism (SQUID), photoluminescence (PL), and field emission (Keithley-237), were measured.

## Results and discussion

**Figure 1:**
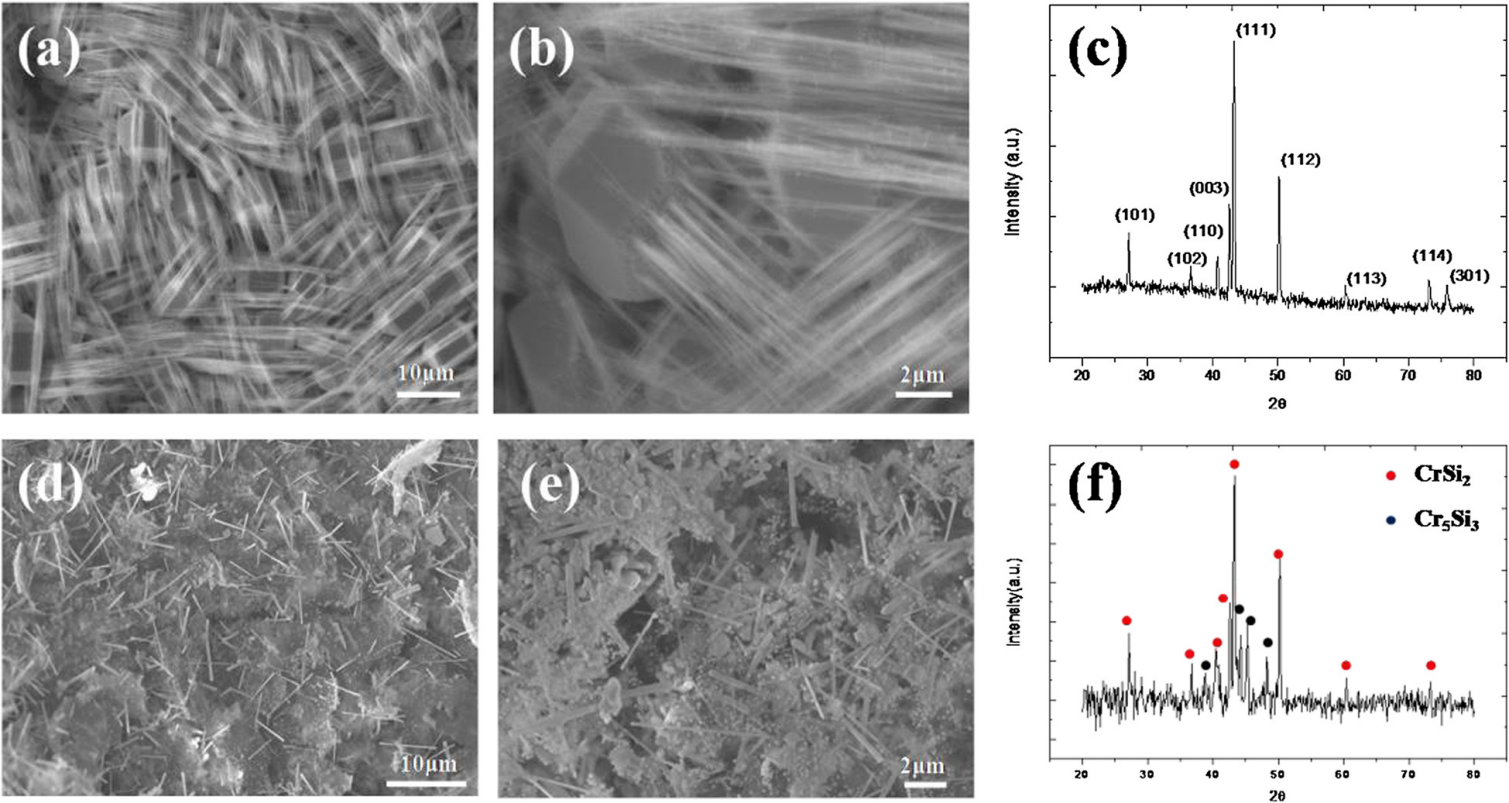
**SEM images and XRD analysis of chromium silicide nanowires. (a)** Low magnification, **(b)** high-resolution SEM images, and **(c)** XRD analysis of CrSi_2_ nanowires grown at 700°C. **(d)** Low magnification, **(e)** high-resolution SEM images and **(f)** XRD analysis of Cr_5_Si_3_ nanowires grown at 750°C with H_2_ atmosphere.

**Figure 2:**
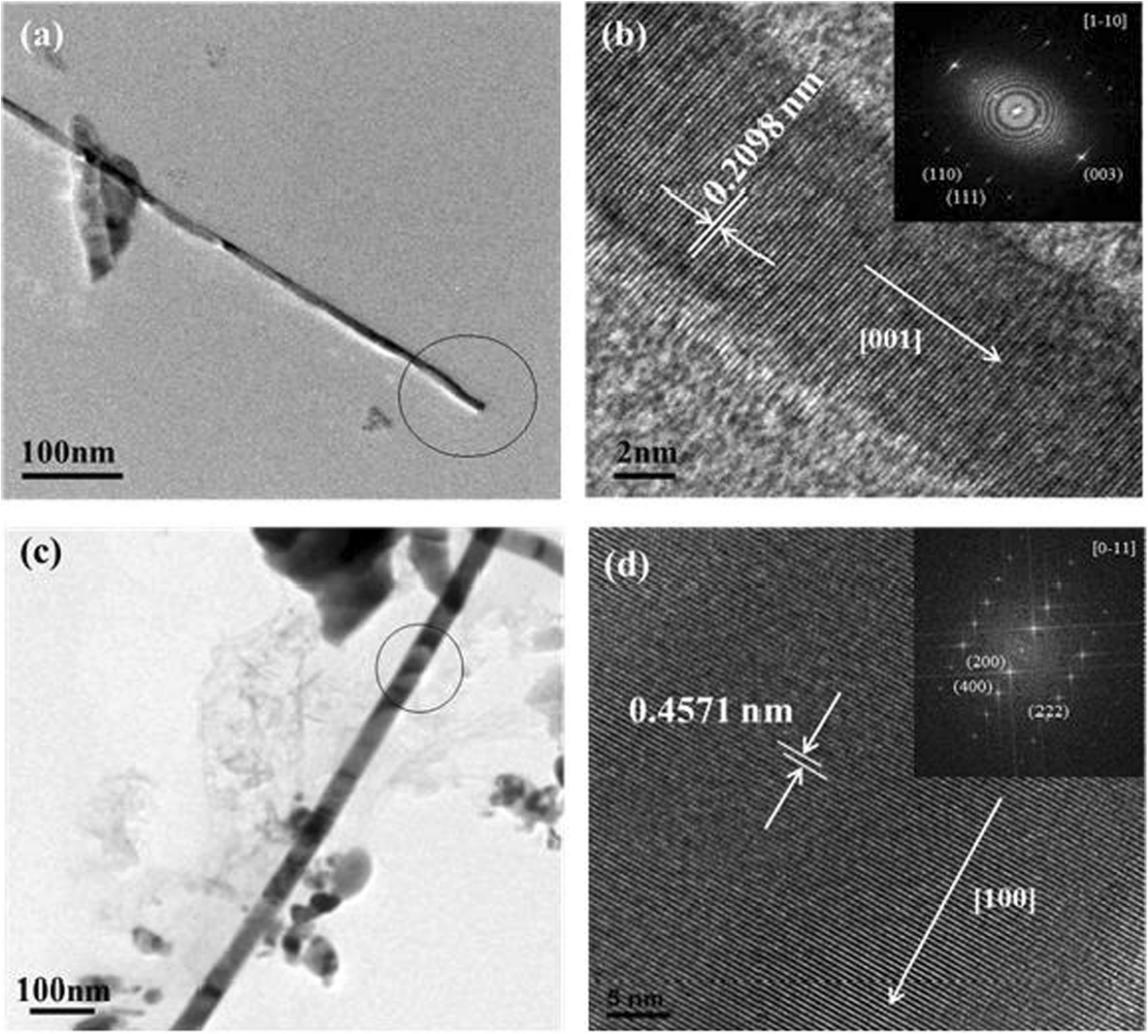
**TEM analysis of chromium silicide nanowires. (a)** Low magnification, **(b)** high-resolution TEM images of CrSi_2_ nanowires grown at 700°C. The inset in **(b)** shows the corresponding fast Fourier transform (FFT) pattern with a zone axis of [1–10]. **(c)** Low magnification, **(d)** high-resolution TEM images of Cr_5_Si_3_ nanowires grown at 750°C. The inset in **(d)** shows the corresponding FFT pattern with a zone axis of [0–11].

In this work, we controlled different parameters to realize how they influence the nanowires’ growth, morphology, and physical properties. With source and substrate at 700°C and the flow gas of 120 sccm, we obtained dense CrSi_2_ nanowires with a length of approximately 20 μm as shown in Figure 1a by chemical vapor deposition. Interestingly, in Figure 1b, the nanowires grew from the particle with almost coherent growth direction and the morphology was rare. XRD analysis in Figure 1c shows (111), (003), and (112) major plane peaks, indicating that the nanowires have a C40 hexagonal structure. The TEM image of Figure 2a shows that the nanowires are 10 to 50 nm in diameter. In Figure 2b, the high-resolution transmission electron microscopy (HRTEM) image and the corresponding fast Fourier transform (FFT) pattern in the inset identifies the materials to be single-crystal CrSi_2_ nanowires of a hexagonal structure with lattice constants, *a* = 0.4428 nm and *c* = 0.6369 nm (JCPDS card no. 35–0781); the growth direction is [001], and the interplanar spacing of plane (003) is 0.2098 nm. Additionally, we tried 750°C with hydrogen as reducing atmosphere and obtained Cr_5_Si_3_ nanowires of approximately 10 μm in length and of a different morphology as shown in Figure 1d. In Figure 1e, we found that the nanowires grew from nanoparticles again. XRD analysis in Figure 1f shows two phases, CrSi_2_ and Cr_5_Si_3_; for further investigation on the atomic structures of the nanowires, we conducted TEM analysis as shown in Figure 2. From the TEM image of Figure 2c, the nanowire was of approximately 80 nm in diameter. The HRTEM image and the corresponding FFT pattern in the inset of Figure 2d confirm that the single-crystal Cr_5_Si_3_ nanowire has a BCT D8m structure with lattice constants, *a* = 0.9165 nm and *c* = 0.4638 nm (JCPDS card no. 51–1357); also, the nanowire is with [100] growth direction, and the interplanar spacing of plane (200) is 0.4571 nm.

**Figure 3:**
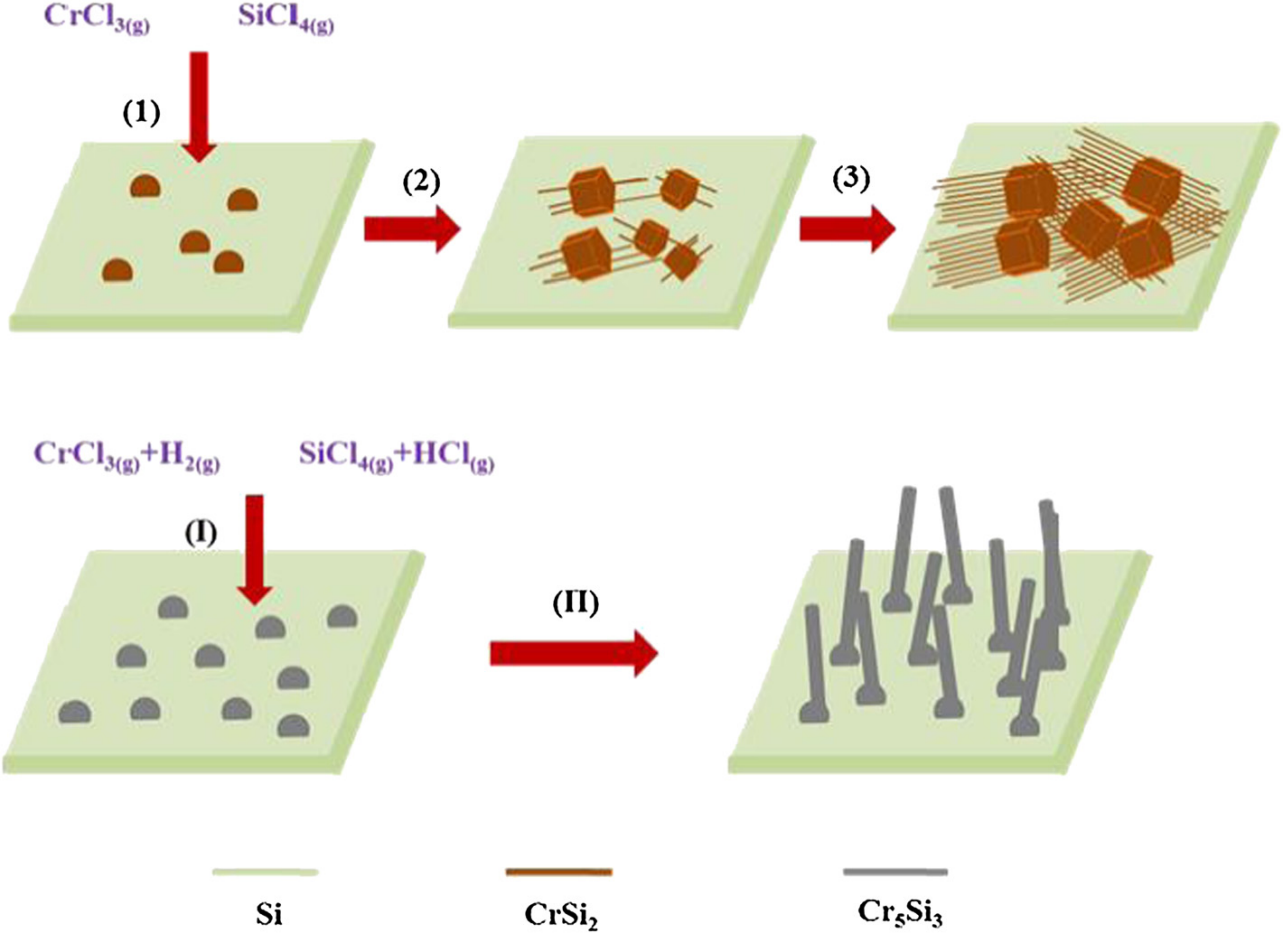
**Schematic illustration of the growth mechanism.** (1) 4CrCl_3(g)_ + 11Si_(s)_ → 4CrSi_2(s)_ + 3SiCl_4(g)_; 4SiCl_4(g)_ + 2CrCl_3(g)_ → 2CrSi_2(l)_ + 11Cl_2(g)_. (2) Growth of CrSi_2_ particles and nanowires. (3) High-density CrSi_2_ nanowires. (I) 10CrCl_3(g)_ + 12Si_(s)_ + 3H_2(g)_ → 2Cr_5_Si_3(s)_ + 6SiCl_4(g)_ + 6HCl_(g)_. (II) Growth of Cr_5_Si_3_ nanowires.

The growth mechanism of the chromium silicide nanowires in this study is interesting. Figure 3 is the schematic illustration of the growth mechanism, showing the proposed growth steps of the CrSi_2_ nanowires. When the system was heated below 700°C, CrCl_3_ · 6H_2_O transformed to CrCl_3_ and H_2_O:

$$ {\mathrm{CrCl}}_3.6{\mathrm{H}}_2{\mathrm{O}}_{\left(\mathrm{g}\right)}\to {\mathrm{CrCl}}_{3\left(\mathrm{g}\right)}+6{\mathrm{H}}_2\mathrm{O} $$

The CrCl_3_ gas molecules then agglomerated on the silicon substrate. As the system temperature reached the reaction temperature, 700°C, CrCl_3_ gas reacted with the silicon substrate to form CrSi_2_ nanoparticles and SiCl_4_ based on step (1) of Figure 3:

$$ 4\mathrm{C}\mathrm{r}\mathrm{C}{\mathrm{l}}_{3\left(\mathrm{g}\right)}+11\mathrm{S}{\mathrm{i}}_{\left(\mathrm{s}\right)}\to 4\mathrm{C}\mathrm{r}\mathrm{S}{\mathrm{i}}_{2\left(\mathrm{s}\right)}+3\mathrm{SiC}{\mathrm{l}}_{4\left(\mathrm{g}\right)}\ T=700{}^{\circ}C $$

The SiCl_4_ product then reacted with CrCl_3(g)_ to form CrSi_2_, following step (2) of Figure 3:

$$ 4{{\mathrm{SiCl}}_4}_{\left(\mathrm{g}\right)}+2{{\mathrm{CrCl}}_3}_{\left(\mathrm{g}\right)}\to 2{\mathrm{CrSi}}_{2\left(\mathrm{l}\right)}+11{{\mathrm{Cl}}_2}_{\left(\mathrm{g}\right)}\ T=700{}^{\circ}C $$

**Figure 4:**
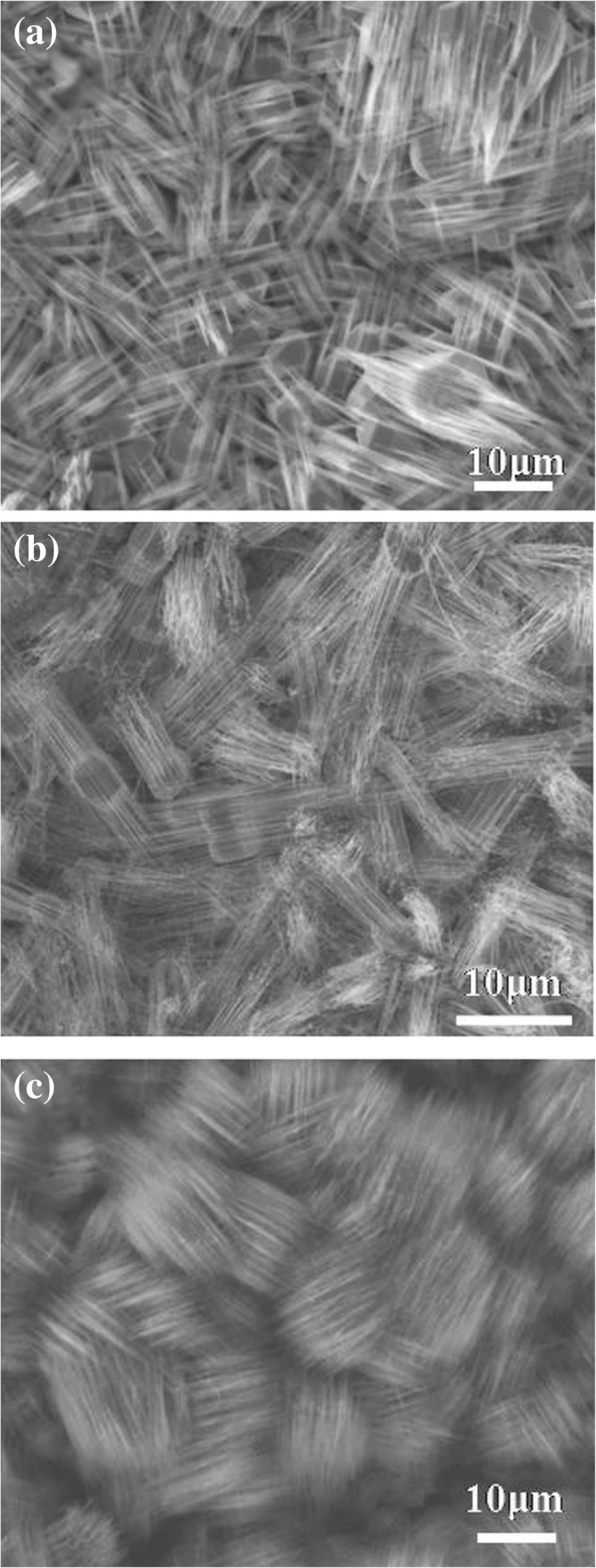
**SEM images of CrSi**_**2**_ **nanowires at different heating times of (a) 1.5, (b) 4, and (c) 12 h, respectively.**

Notably, the CrSi_2_ nanowires precipitated from polygonal particles, and the growth direction seems consistent as shown in Figure 1b. The nanowires and polygonal particles may have the same stacking plane, (003), based on our TEM analysis, and nanowires grew from voids and defects on the surface of any polygonal particles with <001 > growth direction, following step (3) of Figure 3 as shown in a SEM image of Additional file 1: Figure S1. We conducted experiments with the heating times of 1.5, 4, and 12 h at 700°C, obtaining the corresponding results shown in Figure 4a, b, c, respectively. We found nanowires and particles at 1.5 h, more nanowires growing from particles at 4 h, and dense nanowires appearing with buried particles at 12 h, respectively. With a longer duration, more nanowires can overcome the activation energy, successfully nucleate, and grow to be nanowires, contributing to CrSi_2_ nanowires of a high density. According to the observations, we proposed that the mechanism of the nanowire growth is a self-catalytic process.

As the substrate temperature was at 750°C, CrCl_3_ gas reacted with H_2_ gas and the silicon substrate to form Cr_5_Si_3_ nanoparticles, HCl, and SiCl_4_, following step (i) of Figure 3:

$$ 10{\mathrm{Cr}\mathrm{Cl}}_{3\left(\mathrm{g}\right)}+12{\mathrm{Si}}_{\left(\mathrm{s}\right)}+3{\mathrm{H}}_{2\left(\mathrm{g}\right)}\to 2{\mathrm{Cr}}_5{\mathrm{Si}}_{3\left(\mathrm{s}\right)}+6{\mathrm{Si}\mathrm{Cl}}_{4\left(\mathrm{g}\right)}+6{\mathrm{H}\mathrm{Cl}}_{\left(\mathrm{g}\right)}\ T=750{}^{\circ}C $$

The SiCl_4_ also reacted with CrCl_3_ to form CrSi_2_, which is the reason why the XRD analysis shows both CrSi_2_ and Cr_5_Si_3_ phases.

**Figure 5:**
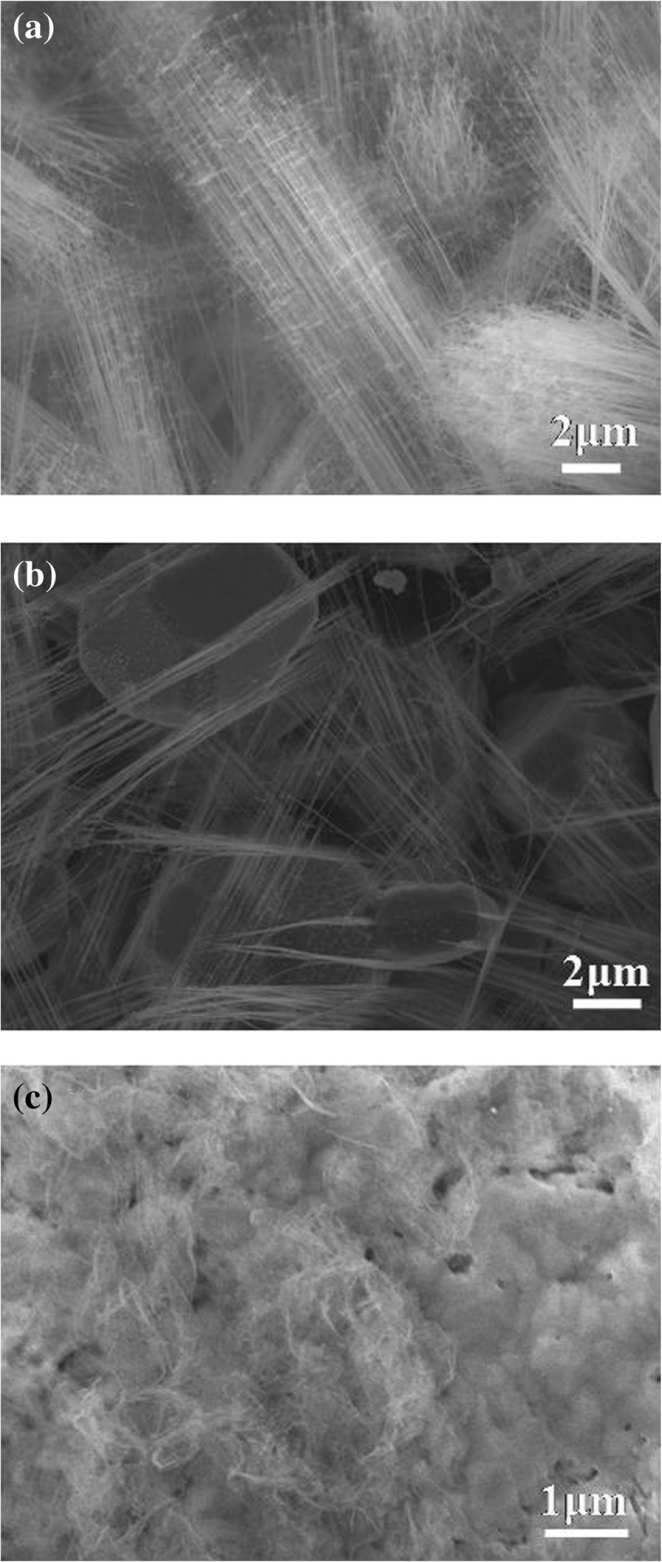
**SEM images of CrSi**_**2**_ **nanowires at different gas flow rates of (a) 60, (b) 120, and (c) 240 sccm, respectively.**

Also, we investigated the influence of the carrier gas flow rate when synthesizing chromium silicide nanowires. We conducted experiments at the gas flow rate of 60, 120, and 240 sccm at 700°C, obtaining the corresponding results shown in Figure 5a, b, c, respectively. It can be found that chromium disilicide nanowires appeared without particles at 60 sccm and with few particles at 120 sccm and that the morphology gradually transformed from nanowires to films at 240 sccm.

The CVD synthesis system can be divided into three sub-systems, which are momentum control system, mass transfer control system, and surface reaction control system. At a lower gas flow rate, mass transfer control system would be the main reaction mechanism, with which gas adsorption and desorption occurred on the Si wafer and fabrication of chromium silicide nanowires was preferred. On the other hand, at a higher gas flow rate, surface reaction control system would be the main reaction mechanism, with which CrCl_3_ reacted on the Si wafer surface by chemical vapor deposition; thus, chromium silicide films appeared.

**Figure 6:**
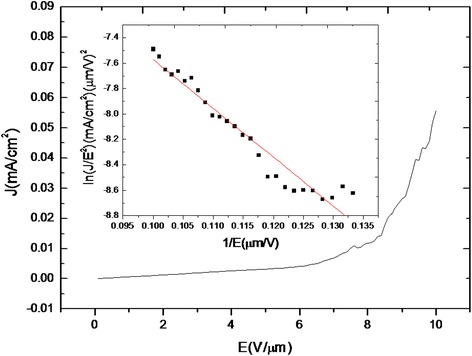
**The field emission measurements of CrSi**_**2**_ **NWs; the inset shows the corresponding ln(J/E** ^**2**^ **)-1/E plot.**

In addition to understanding the growth behaviors of the chromium silicide nanowires, we explored their physical properties. Figure 6 is the field emission measurements for CrSi_2_ NWs, showing the plot of the current density (J) as a function of the applied field (E) with the inset of the ln(J/E^2^)-1/E plot. The sample was measured in a vacuum chamber pump to approximately 10^−6^ Torr. According to the Fowler-Nordheim (F-N) plot and the Fowler-Nordheim equation:

$$ J=\left(A{\mathrm{\ss}}^2{E}^2/\varphi \right)\; \exp \left(-B{\varphi}^{3/2}/\mathrm{\ss}E\right), $$

where *J* is the current density, *E* is the applied electric field, *φ* is the work function, and *A*, *B* are constants, respectively. We put +1,000 V on the sample with a 100-μm spacing between the anode and cathode, and we defined the turn-on field could obtain a current density of 10 μA/cm^2^ and the turn-on field we measured for CrSi_2_ nanowires was 7.5 V/μm. The field enhancement factor *ß* has been calculated to be 1,366 from the slope of ln(*J*/*E*^2^) = ln(*Aß*^2^/*φ*) − *Bφ*^3/2^/*ßE* (for CrSi_2,_*φ* = 3.9 eV [19]), demonstrating that CrSi_2_ NWs are promising emitters. The outstanding field emission properties of CrSi_2_ NWs are attributed to their metallic property and special one-dimensional geometry with a high aspect ratio as compared with those of many other materials.

**Figure 7:**
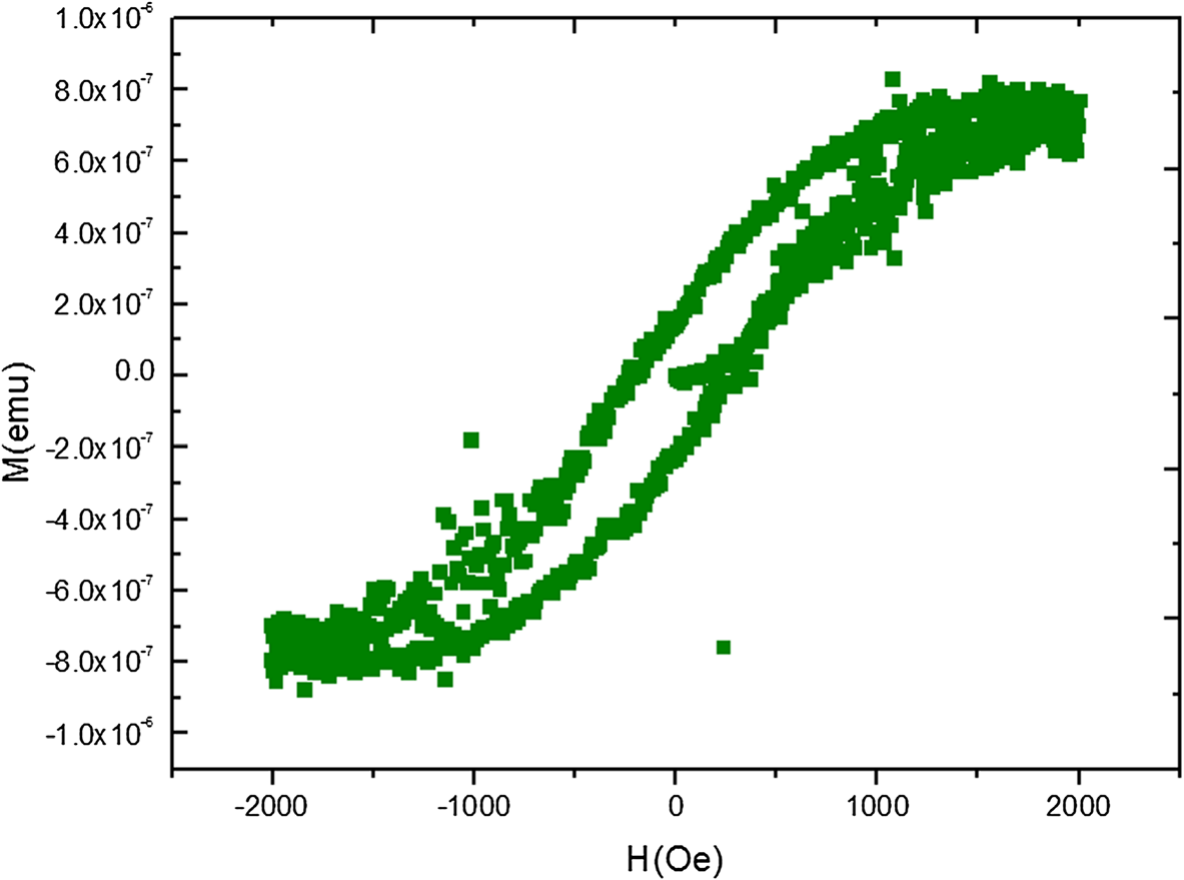
**The magnetism measurements of CrSi**_**2**_ **/SiO**_**x**_ **nanowires.**

On magnetization analysis for chromium disilicide nanowires coated with a silicon oxide layer of a few nanometers in thickness, we prepared samples of 2.5 mm × 2.5 mm with the applied magnetic field of ±3,000 Oe perpendicular to the substrates. Notably, Figure 7 shows that the CrSi_2_/SiO_x_ nanowires grown here were found to be ferromagnetic with the saturation magnetization of 8 × 10^−7^ emu, *M*_R_, remanence, of 2 × 10^−7^ emu, and *H*_C_, coercive force, of about 179 Oe, respectively, which is different from the antimagnetic behavior in CrSi_2_ and SiO_X_. The ferromagnetic characteristic results from the bonding formation between the Si sp hybrid orbitals and the Cr 3d orbitals at the SiOx/CrSi_2_ interface, where the oxygen atoms play an important role, bonding with silicon atoms and making chromium atoms with unpaired electrons, which contributes to ferromagnetism at nanoscale [25].

**Figure 8:**
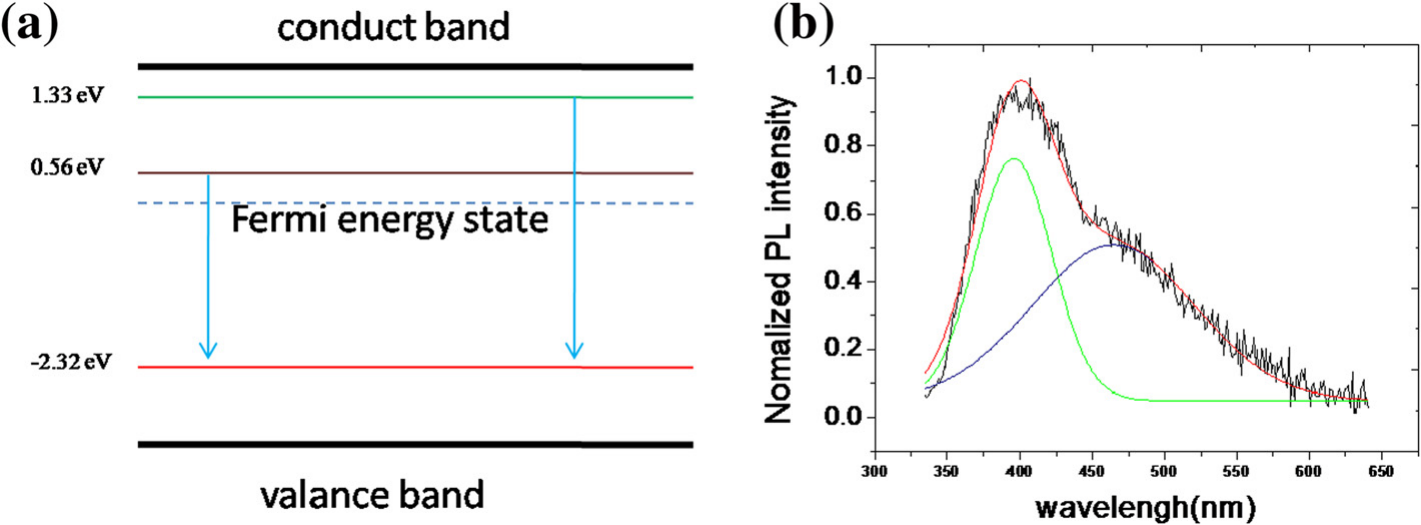
**PL spectrum for the CrSi**_**2**_ **nanowires. (a)** Energy states of CrSi2 bulk. **(b)** Photoluminescence measurements of CrSi2 NWs with Gaussian fitting.

On photoluminescence analysis, Bhamu et al. studied the density of state (DOS) of CrSi_2_ bulk, including 1.33 eV, 0.56 eV above Fermi state, and 2.23 eV under Fermi state [29]. Figure 8b shows our PL spectrum in the visible region for the CrSi_2_ nanowires, where the wide peak was present (red line) and through Gaussian fitting; the other two peaks, 396 nm (green line) and 465 nm (blue line), were calculated. Theoretically, the electron-hole pair recombinations of 1.33 eV, 0.56 eV conduct state to −2.23 eV valance state were 348 and 430 nm for CrSi_2_ bulk. In reality, the difference results from dimension, bulk, and nanowires; as the particle size reduces, wider bandgap light absorption band will move to shorter wavelengths, which is so-called blueshift [30]; however, there may be redshift as well; as the particle size decreases, the internal stress will increase, causing changes in the band structure [31] and the electron wave function overlap to increase the energy gap narrowing [32]; if the redshift factor is larger than the blueshift, then we will see redshift phenomenon, which is the case here.

## Conclusions

In this study, using a CVD method, we have successfully synthesized chromium silicide nanowires of two phases with unique morphologies. Effects of some processing parameters, including the temperature, gas flow rate, and heating time, were investigated; for example, the growth of chromium disilicide nanowires were influenced by CrSi_2_ vapor supersaturation, CrSi_2_ vapor formation rate, and CVD control system. Also, the growth mechanism has been proposed. Field emission and photoluminescence measurements demonstrate that the CrSi_2_ nanowires are potential field-emitting and photovoltaic materials with a low turn-on field. Additionally, the magnetic property measurements for the CrSi_2_/SiO_x_ nanowires, showing a ferromagnetic characteristic, demonstrate promising applications for magnetic storage and biological cell separation.

## Additional file

Additional file 1: Figure S1. SEM image of CrSi_2_ nanowires growing from voids and defects on the surface of silicide particles at 700°C.
