# Supplementary material for: Single-crystalline chromium silicide nanowires and their physical properties
Source: Nanoscale Res Lett. 2015 Feb 6;10:50. doi: 10.1186/s11671-015-0776-8 (PMC4385120; doi:10.1186/s11671-015-0776-8)
Supplement: Additional file 1: Figure S1. — SEM image of CrSi2 nanowires growing from voids and defects on the surface of silicide particles at 700°C. [file 11671_2015_776_MOESM1_ESM.pdf]

Single Crystalline Chromium Silicide Nanowires and Their Physical Properties

Han-Fu Hsu[1], Ping-Chen Tsai[1] and Kuo-Chang Lu[1,2]*

[1] Department of Materials Science and Engineering, National Cheng Kung University, Tainan 701, Taiwan

[2] Center for Micro/Nano Science and Technology, National Cheng Kung University, Tainan 701, Taiwan

*Corresponding author

Email: gkclu@mail.ncku.edu.tw

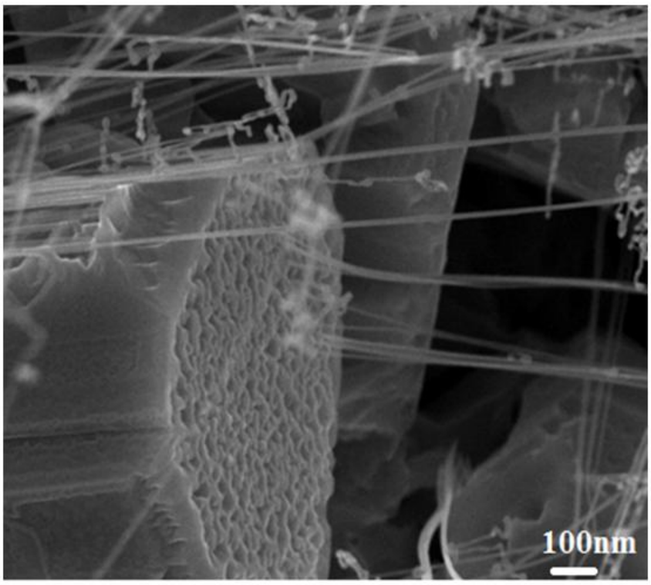

Figure S1 SEM image of $CrSi_2$ nanowires growing from voids and defects on the surface of silicide particles at 700℃.
